# Development and validation of identification algorithms for five autoimmune diseases using electronic health records: a retrospective cohort study in China

**DOI:** 10.3389/fimmu.2025.1541203

**Published:** 2025-04-10

**Authors:** Junting Yang, Yunxiao Wu, Jinxin Guo, Xiaoxuan Wang, Xin Gao, Xin Chen, Mengdi Zhang, Jin Yang, Zuojing Liu, Yan Liu, Zhike Liu, Siyan Zhan

**Affiliations:** ^1^ Department of Epidemiology and Biostatistics, School of Public Health, Peking University, Beijing, China; ^2^ Key Laboratory of Epidemiology of Major Diseases (Peking University), Ministry of Education, Beijing, China; ^3^ Department of Endocrinology and Metabolism, Peking University Third Hospital, Beijing, China; ^4^ Department of Gastroenterology, Peking University Third Hospital, Beijing, China; ^5^ Department of Hematology, Peking University Third Hospital, Beijing, China; ^6^ Research Center of Clinical Epidemiology, Peking University Third Hospital, Beijing, China; ^7^ Center for Intelligent Public Health, Institute for Artificial Intelligence, Peking University, Beijing, China

**Keywords:** Hashimoto's thyroiditis, inflammatory bowel disease (IBD), primary immune thrombocytopenia, rheumatoid arthritis (RA), type 1 diabetes (T1D), computable phenotype, algorithms, electronic health records (EHR)

## Abstract

**Objective:**

This study aims to assess the identification algorithms for five autoimmune diseases—Hashimoto’s thyroiditis, inflammatory bowel disease (IBD), primary immune thrombocytopenia (ITP), rheumatoid arthritis (RA), and type 1 diabetes (T1D)—using the Yinzhou Regional Health Information Platform (YRHIP) in China.

**Methods:**

Diagnostic data was extracted from YRHIP’s population registry (2010-2021), combining ICD-10 codes and Chinese medical terminology from outpatient, inpatient, and discharge records. Algorithms were validated through chart reviews, adhering to global clinical guidelines. Cases were adjudicated using electronic case report forms. We evaluated algorithm performance based on sensitivity and positive predictive value (PPV), with a 70% PPV threshold for optimization.

**Results:**

Among all reviewed cases, we identified 136 cases for Hashimoto’s thyroiditis, 65 for IBD, 76 for ITP, 130 for RA, and 43 for T1D. Algorithm performance varied across diseases: the final algorithm for Hashimoto’s thyroiditis achieved optimal accuracy (sensitivity 97.44%, PPV 98.28%), followed by RA (sensitivity 100.00%, PPV 76.92%). Algorithms for IBD and ITP required synthesis of multiple data sources to achieve acceptable performance (IBD: sensitivity 79.66%, PPV 70.15%; ITP: sensitivity 62.50%, PPV 70.00%). For T1D, the final algorithm utilizing both admission and outpatient records yielded satisfactory results (sensitivity 84.09%, PPV 74.00%).

**Conclusions:**

This study presents the first validated algorithms for identifying autoimmune diseases using EHR data in China, demonstrating satisfactory performance (PPV >70%) across all diseases. Our findings demonstrate that a combination of data sources is crucial for accurate case identification in complex autoimmune conditions, providing an important methodological foundation for future real-world studies in Chinese populations.

## 1 Introduction

Autoimmune diseases (AD) constitute a group of conditions where the immune system mistakenly attacks the body’s own tissues, leading to severe tissue/organ destruction or even fatal outcomes. Factors such as genetics, environmental changes, and vaccinations have been implicated in their etiology (1). Through rigorous epidemiological studies, it has now been shown that autoimmune diseases affect 3-10% of the global population (2, 3), suggesting an urgent demand for the control of these diseases. A study utilizing the Clinical Practice Research Datalink (CPRD) database explored the prevalence of 19 common autoimmune diseases, noting an increase from 7.7% in 2000 to 11.0% in 2019 (4). Further monitoring of the burden of these diseases in different regions and countries using high-quality real-world databases is essential.

As the demand for data-driven healthcare continues to rise, real-world studies (RWS) have become increasingly important in the context of regulatory decision-making. Nevertheless, a major challenge faced by RWS is the issue of data quality (5). The U.S. Food and Drug Administration’s (FDA) Framework for Real-World Evidence Program underscores the necessity for reliable and relevant real-world data (RWD), with the accuracy of key variables being pivotal to data reliability (6). In the contexts of disease burden estimation, associative analyses, and vaccine safety surveillance, it is imperative to reduce biases stemming from misclassification. To maximize the efficacy of these studies, it is necessary to ensure the validity of outcome variables and possess an in-depth understanding of the definition algorithms (7, 8).

To address these challenges in disease identification and classification, computable phenotypes have emerged as a promising solution. These are meaningful health or disease characteristics extracted from raw data through data analysis and computational models (9). These algorithms include one or more structured data elements, such as ICD codes, laboratory test results, or medication prescriptions, which can identify specific disease risk factors or subtypes from electronic health records (EHR). This approach provides high-fidelity, personalized, and interpretable phenotype estimations, enabling researchers to extract valuable information from vast medical datasets, thereby enhancing diagnostic and therapeutic efficiency (10). Several studies have been conducted to develop and validate computable phenotypes for diseases such as hypertension, pulmonary hypertension, and adverse drug reactions in real-world databases (11, 12). However, there are few similar studies in China (13), and none for autoimmune diseases.

The Yinzhou Regional Health Information Platform (YRHIP) in Ningbo, China, has been validated as a reliable and robust real-world database (14, 15). It encompasses various types of RWD, such as EHR, registries, and electronic medical records (EMR). These data are converted into a structured format and linked to a unique national identifier or healthcare identifier. YRHIP has been extensively utilized in fields such as infectious disease surveillance, drug safety evaluation, chronic disease management, generating numerous high-quality studies and yielding significant societal benefits. Furthermore, we emphasize that assessing the accuracy of AD diagnostic records in the YRHIP is crucial for the active post-market surveillance of the HPV vaccine safety (16). In summary, this study aims to validate the performance of algorithms used to identify five specific ADs utilizing the YRHIP. The findings are intended to provide valuable insights for related real-world studies.

## 2 Methods

### 2.1 Data sources and study population

The YRHIP is a comprehensive electronic health information system established in 2005 by the Yinzhou District Center for Disease Control and Prevention. It is designed to enhance routine primary care services offered by local general practitioners. YRHIP retains the EHRs of the population in Yinzhou District, Ningbo, Zhejiang Province, which encompasses 976,409 permanent residents with valid healthcare coverage as identified by the end of 2019 (17). This database platform has progressively incorporated information from public health surveillance, hospital health information systems, maternal and child healthcare, immunizations, population screening, disease management, and other healthcare services. Since 2009, YRHIP has covered nearly all healthcare-related activities for residents from birth to death (16).

This study utilized a retrospective cohort design, utilizing the YRHIP database to compile registration information for all permanent residents documented in the EHRs maintained within the YRHIP. Data collection spanned from January 2010 to June 2021. The study specifically focused on healthcare visitations related to five ADs of interests: Hashimoto’s thyroiditis, inflammatory bowel disease (IBD), primary immune thrombocytopenia (ITP), rheumatoid arthritis (RA), and type I diabetes (T1D). Mortality data were included to appropriately address censoring.

Inclusion criteria for the cohort were: a. All permanent residents with records in YRHIP from January 2010 to June 2021. Permanent residents are defined as those who have resided in the area for more than six months and have been registered in the EHR of YRHIP; b. Diagnosis during the observation period with one of the five ADs of interest. Exclusion criteria for the cohort were: a. Missing health record registration date in YRHIP; b. Missing unique personal identification code.

### 2.2 Identification of autoimmune diseases and incident cases

Within the cohort, cases were identified using disease diagnostic terms and/or International Classification of Diseases (ICD-10) codes (**Table 1**). We identified potential patients by assessing any disease diagnoses in outpatient records, emergency records, admission records, discharge records, and inpatient records. To ensure the integrity of clinical records used for chart review, this study focused exclusively on “incident cases” of admission for validation purposes. Incident cases included those patients who were admitted upon their initial diagnosis and patients admitted within one month following an outpatient diagnosis. A one-year washout period was applied, consistent with previous studies. Non- hospitalized patients were excluded due to insufficient data for validation. Notably, T1D was identified using terms such as “type I diabetes,” “insulin-dependent diabetes,” and ICD-10 code “E10”.

**Table 1:** Clinical guidelines and diagnostic criteria of autoimmune diseases.

Autoimmune diseases (English and Chinese)	ICD10	Reference guidelines	Diagnostic criteria
Hashimoto’s thyroiditis	E06.3	the Chinese guidelines for the diagnosis and treatment of thyroiditis (18)	“2 and 5 and 6” and (or) “1 and 3 and 4” of Level 1 of certainty ^a^
Inflammatory bowel disease	K51, K50	1. the Consensus on the Diagnosis and Treatment of Inflammatory Bowel Disease (Beijing, 2018) (19) 2. the World Gastroenterology Organization Practice Guidelines for the diagnosis and management of IBD in 2010 (20)	Level 1 of certainty
Primary immune thrombocytopenia	D69.3, D69.4	1. the Brighton Collaboration’s guidelines for Thrombocytopenic Purpura (21) 2. the Pediatric Primary Immune Thrombocytopenia clinical Guidelines (2019 Edition) (22)	Level 1 of certainty
Rheumatoid arthritis	M0[56]\.[389]	1. The American Rheumatism Association 1987 revised criteria for the classification of rheumatoid arthritis (23) 2. 2010 ACR/EULAR rheumatoid arthritis classification criteria: an American College of Rheumatology/European League Against Rheumatism collaborative initiative (24)	Meet one of the two criteria from reference guidelines ^b^
Type I diabetes	E10	the Chinese Guidelines for the Diagnosis and Treatment of Type 1 Diabetes Mellitus (2013 edition) (25)	Level 1 of certainty

Specific details on clinical examinations and symptoms for the diagnostic criteria of each disease are provided in the electronic case report forms for each disease, available in Appendix 1. a. Considering that the definitive criteria for Hashimoto’s thyroiditis involve pathological examinations such as biopsy, which are difficult to obtain, meeting certain essential clinical criteria is sufficient for diagnosis. b. The standards of the two clinical guidelines are not entirely identical, but their diagnostic capabilities are equivalent; therefore, meeting the criteria of either one is acceptable.

### 2.3 Adjudication of autoimmune diseases and algorithms

In this study, we employed a chart review method for case verification, engaging two clinical physicians (at or above the department head level) to independently assess the cases based on clinical guidelines and standards. They provided a conclusion of “yes,” “no,” or “undeterminable,” and also indicated whether the case was a previous one. Situations where insufficient data prevented a definitive conclusion were categorized as “undeterminable.” If there was a disagreement between the two physicians, a third clinical physician participated in a discussion to reach a consensus.

Data collection was conducted using a standardized electronic case report form (eCRF), developed with reference to international clinical guidelines (**Table 1**), which were reviewed and confirmed by clinical experts at the department head level. The eCRF varied slightly between different diseases and primarily included definitive grading of diseases and suspected case information forms. The definitive grading incorporated criteria such as symptom manifestation, auxiliary examination results, and surgical outcomes. The case information form gathered essential data for determining definitive grading, including visiting information, diagnostic information and clinical symptoms, medical examination and laboratory testing, and the reviewed grading for suspected cases. These eCRFs were completed by trained clinical physicians or personnel with relevant medical expertise. (Appendix 1)

The identification algorithm was constructed using diagnostic terms and ICD-10 codes, with a screening condition of positive predictive value (PPV)≥70% to finalize the algorithm with the optimal sensitivity (13). The algorithm structure was broadly as follows (specific algorithms for each autoimmune disease are detailed in **Table 2**): a. The ICD-10 code related to the disease of interest; b. The Chinese terminology related to the disease of interest; c. Either the ICD-10 code or the Chinese terminology related to the disease of interest.

**Table 2:** Performance of algorithms/computable phenotype for identification of autoimmune diseases.

Autoimmune diseases	Algorithms	Valid Cases	Confirmed Cases	SEN (95%CI)	PPV (95%CI)
Hashimoto’s thyroiditis					
	1: Diagnosis of admission records	13	13	11.11 (5.42, 16.81)	100.00 (100.00,100.00)
	2: ICD10 of admission records	3	3	2.56 (0.00, 5.43)	100.00 (100.00,100.00)
	3: Diagnosis of discharge records	116	114	97.44 (94.57,100.00)	98.28 (95.91,100.00)
	4: ICD10 of discharge records	98	97	82.91 (76.08,89.73)	98.98 (96.99,100.00)
	5: Diagnosis of outpatient records	17	17	14.53 (8.14, 20.92)	100.00(100.00,100.00)
	6: ICD10 of outpatient records	17	17	14.53 (8.14, 20.92)	100.00(100.00,100.00)
	Final algorithms: 1-6	116	114	97.44(94.57,100.00)	98.28(95.91,100.00)
Inflammatory bowel disease					
	1: Diagnosis of admission records: “Crohn’s disease”	6	6	10.17 (2.46,17.88)	100.00 (100.00,100.00)
	2: ICD10 of admission records: K50	0	0	NA	NA
	3: Diagnosis of admission records: “ulcerative colitis”	30	25	42.37 (29.76,54.98)	83.33 (70.00,96.67)
	4: ICD10 of admission records: K51	4	3	5.08 (0.00,10.69)	75.00 (32.57,100.00)
	5: Diagnosis of discharge records: “Crohn’s disease”	18	13	22.03 (11.46,32.61)	72.22 (51.53,92.91)
	6: ICD10 of discharge records: K50	17	12	20.34 (10.07,30.61)	70.59 (48.93,92.25)
	7: Diagnosis of discharge records: “ulcerative colitis”	67	35	59.32 (46.79,71.86)	52.24 (40.28,64.20)
	8: ICD10 of discharge records: K51	69	35	59.32 (46.79,71.86)	50.72 (38.93,62.52)
	9: Diagnosis of outpatient records: “Crohn’s disease”	19	16	27.12 (15.77,38.46)	84.21 (67.81,100.00)
	10: ICD10 of outpatient records: K50	19	16	27.12 (15.77,38.46)	84.21 (67.81,100.00)
	***11: Diagnosis of outpatient records: “ulcerative colitis	11	11	18.64 (8.71,28.58)	100.00 (100.00,100.00)
	***12: ICD10 of outpatient records: K51	43	32	18.64 (8.71,28.58)	74.42 (61.38,87.46)
	Final algorithms: 1, 3, 5, 6, 9-12	67	47	79.66 (69.39,89.93)	70.15 (59.19,81.11)
Primary immune thrombocytopenia					
	1: Diagnosis of admission records: “immune thrombocytopenia”	4	4	7.14 (0.40,13.89)	100.00 (100.00,100.00)
	2: ICD10 of admission records: D69.3	2	2	3.57 (0.00,8.43)	100.00 (100.00,100.00)
	3: Diagnosis of admission records: “idiopathic thrombocytopenia”	4	3	5.36 (0.00,11.25)	75.00 (32.57,100.00)
	4: ICD10 of admission records: D69.4	0	0	NA	NA
	5: Diagnosis of discharge records: “immune thrombocytopenia”	29	21	37.50 (24.82,50.18)	72.41 (56.15,88.68)
	6: ICD10 of discharge records: D69.3	33	23	41.07 (28.19,53.96)	69.70 (54.02,85.38)
	7: Diagnosis of discharge records: “idiopathic thrombocytopenia”	8	5	8.93 (1.46,16.4)	62.50 (28.95,96.05)
	8: ICD10 of discharge records: D69.4	12	5	8.93 (1.46,16.4)	41.67 (13.77,69.56)
	9: Diagnosis of outpatient records: “immune thrombocytopenia”	31	22	39.29 (26.49,52.08)	70.97 (54.99,86.95)
	**10: ICD10 of outpatient records: D69.3	23	19	33.93 (21.53,46.33)	82.61 (67.12,98.10)
	**11: Diagnosis of outpatient records: “idiopathic thrombocytopenia”	10	8	14.29 (5.12,23.45)	80.00 (55.21,100.00)
	12: ICD10 of outpatient records: D69.4	6	5	8.93 (1.46,16.4)	83.33 (53.51,100.00)
	Final algorithms: 1-3, 5, 9-12	50	35	62.50 (49.82,75.18)	70.00 (57.3,82.7)
Rheumatoid arthritis					
	1: Diagnosis of admission records: “rheumatoid arthritis”	17	15	50.00 (32.11,67.89)	88.24 (72.92,100.00)
	2: ICD10 of admission records: M0[56]\.[389]	2	2	6.67 (0.00,15.59)	100.00 (100.00,100.00)
	3: Diagnosis of discharge records: “rheumatoid arthritis”	39	30	100.00 (100.00,100.00)	76.92 (63.70,90.15)
	4: ICD10 of discharge records: M0[56]\.[389]	36	29	96.67 (90.24,100.00)	80.56 (67.63,93.48)
	5: Diagnosis of outpatient records: “rheumatoid arthritis”	30	25	83.33 (70,96.67)	83.33 (70,96.67)
	6: ICD10 of outpatient records: M0[56]\.[389]	29	24	80.00 (65.69,94.31)	82.76 (69.01,96.51)
	Final algorithms: 1-6	39	30	100.00 (100.00,100.00)	76.92 (63.70,90.15)
Type I diabetes					
	1: Diagnosis of admission records: “Type I diabetes”	13	11	25.00 (12.21,37.79)	84.62 (65,100.00)
	2: Diagnosis of admission records: “autoimmune diabetes”	1	1	2.27 (0.00,6.68)	100.00 (100.00,100.00)
	3: ICD10 of admission records: E10	2	1	2.27 (0.00,6.68)	50.00 (0.00,100.00)
	4: Diagnosis of admission records: E10 & “diabetic ketosis”	2	1	2.27 (0.00,6.68)	50.00 (0.00,100.00)
	5: Diagnosis of discharge records: “Type I diabetes”	44	31	70.45 (56.97,83.94)	70.45 (56.97,83.94)
	6: Diagnosis of discharge records: “autoimmune diabetes”	0	0	NA	NA
	7: ICD10 of discharge records: E10	0	0	NA	NA
	8: Diagnosis of discharge records: E10 & “diabetic ketosis”	218	38	86.36 (76.22,96.50)	17.43 (12.4,22.47)
	**9: Diagnosis of outpatient records: “Type I diabetes”	20	14	31.82 (18.06,45.58)	70.00 (49.92,90.08)
	**10: ICD10 of outpatient records: E10	14	13	29.55 (16.06,43.03)	92.86 (79.37,100.00)
	**11: Diagnosis of outpatient records: “autoimmune diabetes”	2	2	4.55 (0.00,10.7)	100.00 (100.00,100.00)
	12: Diagnosis of outpatient records: (“Type I diabetes” or E10) & “diabetic ketosis”	15	14	31.82 (18.06,45.58)	93.33 (80.71,100.00)
	Final algorithms: 1, 2, 5, 10, 11, 12	50	37	84.09 (73.28,94.90)	74.00 (61.84,86.16)

Valid cases: cases identified by the algorithm. Confirmed cases: cases identified by the algorithm and validated as confirmed through chart review. Hashimoto’s diagnosis: Hashimoto/Chiomoto, lymphocytic goiter. Hashimoto ICD10: E06.3. Hashimoto’s final algorithm: The above algorithm is arbitrary. **: 2 or more records. ***: 3 or more records. $SEN=\frac{\text{Confirmed cases}}{\text{True cases}}\times 100\%$ ; $PPV=\frac{\text{Confirmed cases}}{\text{Valid cases}}\times 100\%$ ; Total number: The total number of cases identified using disease diagnostic terminology or ICD10. Ture cases: The number of patients confirmed as cases through case verification (gold standard).

### 2.4 Statistical analysis

The performance of each identification algorithm was assessed by determining its sensitivity, and PPV, with 95% confidence intervals calculated using a binomial distribution. Categorical variables were expressed as frequencies (percentages), continuously normally distributed variables as mean ± standard deviation, and non-normally distributed continuous variables as medians and interquartile ranges. Statistical analyses were conducted using SAS^®^ software (version 9.4; SAS Institute Inc., Cary, NC, USA).

## 3 Results

### 3.1 Case extraction and adjudication

The comprehensive extraction from the YRHIP yielded a total of 136 cases for Hashimoto’s thyroiditis, 65 for IBD, 76 for ITP, 130 for RA, and 43 for T1D, with all cases meeting the criteria for further analysis without any being classified as indeterminate. Among these, the distribution of previous cases, eligible cases, and true cases varied by disease, reflecting the complexities in case identification within our retrospective cohort study (**Table 2**).

### 3.2 Performance of algorithms to identify autoimmune diseases

The performance assessment results of the identification algorithms were presented in **Table 2**. Overall, the table illustrated the varying sensitivity and PPV of the algorithms for different autoimmune diseases, with a notable emphasis on the importance of combining ICD-10 codes with diagnostic terms to achieve higher accuracy.

For Hashimoto’s thyroiditis, the algorithm utilizing both ICD-10 codes and diagnostic terms demonstrated the highest accuracy, with a sensitivity of 97.44% (95%CI: 94.57%, 100.00%) and a PPV of 98.28% (95%CI: 95.91%, 100.00%). This indicated that the combined approach was highly effective in identifying true cases of the disease without a significant loss in PPV. Notably, the use of ICD-10 “E06.3” alone resulted in a sensitivity of 11.11% and a PPV of 100.00%, highlighting the limitations of using a single identifier. The final algorithm, which included multiple data sources, significantly improved sensitivity while maintaining a high PPV.

For IBD, the algorithm using outpatient diagnostic terms alone showed a high PPV of 100.00% but a low sensitivity of 18.64%. Conversely, the discharge diagnosis algorithm had a sensitivity of 59.32% and a PPV of 52.24%. The final algorithm, which combined both outpatient and inpatient data, achieved a sensitivity of 79.66% (95% CI: 69.39%, 89.93%) and a PPV of 70.15% (95% CI: 59.19%, 81.11%), reflecting the complexity of diagnosing IBD and the need for multidimensional evidence.

The ITP algorithm using only outpatient diagnostic terms showed a PPV of 100% but a sensitivity below 20%. The final algorithm, which synthesized multiple data sources, achieved a sensitivity of 62.50% (95% CI: 49.82%, 75.18%) and a PPV of 70.00% (95% CI: 57.30%, 82.70%), demonstrating that combining various data sources was necessary to improve the identification of ITP cases.

In the case of RA, the discharge diagnosis ICD-10 code algorithm showed a sensitivity of 100.00% and a PPV of 76.92% (95% CI: 63.70%, 90.15%), which was identical to the final algorithm’s performance. This suggested that the inclusion of discharge diagnosis ICD-10 codes was sufficient for accurate identification of RA cases.

For T1D, the discharge diagnosis “E10 & diabetic ketoacidosis” algorithm had the highest sensitivity at 86.36% (95% CI: 76.22%, 96.50%) but a very low PPV, leading to its exclusion from the final algorithm. The final algorithm, requiring at least two outpatient records, resulted in a PPV of 74.00% (95% CI: 61.84%, 86.16%), demonstrating the need for multiple diagnostic records to ensure accurate identification of T1D cases.

## 4 Discussion

This is the first validation study and evaluation of identification algorithms for autoimmune diseases using EHR in China. Using the 10-year cohort data from YRHIP, we evaluated the performance of diagnostic terminology, ICD-10 codes, and related algorithms for five autoimmune diseases, with a PPV threshold of 70% to optimize sensitivity. The final algorithms demonstrated varying performance, with Hashimoto’s thyroiditis and rheumatoid arthritis achieving excellent accuracy through single diagnostic terms or ICD-10 codes (PPV>70%), while diseases like ITP required more complex combinations yet still showed limited sensitivity.

### 4.1 Algorithm performance and optimization

Our evaluation revealed varying algorithm performance across different autoimmune diseases. While HT achieved the highest accuracy (sensitivity 97.44%, PPV 98.28%), followed by RA (sensitivity 100%, PPV 76.92%), IBD (sensitivity 79.66%, PPV 70.15%), ITP (sensitivity 62.50%, PPV 70.00%), and T1D (sensitivity 84.09%, PPV 74.00%), most algorithms except HT demonstrated lower performance compared to international studies. This discrepancy can be attributed to several key factors. Most notably, established studies have employed more sophisticated algorithm construction approaches. The value of synthesizing multidimensional data is well-documented. For instance, Klompas et al (26) achieved remarkable accuracy in T1D identification (sensitivity 100.00%, 95% CI 96.00%-100.00%; validation cohort: sensitivity 97.00%, PPV 88.00%) by utilizing laboratory values, including plasma C-peptide and autoantibody levels, alongside diagnostic codes and prescription data. Similarly, RA algorithms combining specialist assessments with disease-specific medications reached PPV of 93.60% (27). In IBD studies, the Korean group reported superior performance (sensitivity 94.00%, PPV 97.90%) through therapeutic data synthesis (28), while our results (sensitivity 79.66%, PPV 70.15%) more closely resembled the Ontario study that relied solely on diagnostic information (sensitivity 79.40%) (28). The value of multiple diagnostic records has also been well established. Studies have shown that requiring two or more visits within six months improved ITP identification (PPV 93.00%) (29), with comparable benefits observed in IBD (30) and RA (31) algorithms. Despite these limitations in data dimensions, we achieved modest improvements through strategic data synthesis, combining outpatient, inpatient, and discharge records. This approach notably enhanced disease identification: for IBD, while single outpatient records showed perfect PPV but limited sensitivity (18.64%), our comprehensive algorithm improved sensitivity to 79.66%. Similar patterns emerged for ITP, where initial high PPV (82.61%-100.00%) but low sensitivity (<40%) improved to 62.50% after combining multiple data sources. Population characteristics further influenced algorithm performance. Previous T1D studies focused primarily on younger cohorts (<20 years), where T1D comprises ≥85% of diabetes cases, achieving higher accuracy (PPV 85.50% (32), 89.30% (33)). Our inclusion of the full age spectrum, particularly adults over 30 where type 2 diabetes predominates, introduced additional diagnostic challenges.

Recent advances in algorithm optimization have incorporated artificial intelligence and machine learning methodologie^s^ (34). Notable achievements include convolutional neural network models for RA radiographic assessment (sensitivity 95.00%, specificity 94.00%) (35), and computer-aided diagnostic systems utilizing ultrasound features for HT detection (sensitivity 82.80%, PPV 88.90%) (36). Computational phenotyping approaches continue to evolve, as demonstrated by Zhou et al.’s EHR-based machine learning system achieving 92.29% accuracy in RA identification (37). These advanced methodologies, while promising, face implementation challenges including data accessibility, interpretability limitations, and generalizability constraints. Our structured data-based approach, though yielding moderate performance metrics, offers advantages in transparency and reproducibility.

Outcome validation and algorithm refinement fundamentally aim to address misclassification bias, with varying performance requirements across applications. Pharmacovigilance and vaccine safety studies require high sensitivity for comprehensive case capture, while risk assessment research may prioritize high PPV combined with nested case-control designs. Recent developments include quantitative bias analysis (QBA) for misclassification adjustment (38), with Newcomer et al. emphasizing exposure-stratified PPV assessment (39). Our algorithm selection process balanced these considerations, prioritizing practical utility despite sensitivity limitations in some disease categories.

It is critical to acknowledge the potential impacts of false positives and false negatives. False positives may increase healthcare utilization by triggering unnecessary diagnostic evaluations or overtreatment, thereby escalating medical costs. Conversely, false negatives risk missing individuals with genuine healthcare needs, potentially leading to delayed diagnoses and adverse clinical outcomes (40). However, the cumulative effects of these errors on downstream research and clinical decisions are highly context-dependent. Their interpretation requires careful consideration of the specific application scenarios to avoid overgeneralization. For instance: In disease burden estimation, a high false-positive rate could artificially inflate prevalence estimates, distorting public health prioritization (41). For vaccine/drug safety surveillance, tolerating a controlled level of false positives might enhance sensitivity in early signal detection, albeit at the cost of specificity (42). In comparative effectiveness or risk assessment studies, adjustments using predictive values (e.g., PPV) or sensitivity analyses are essential to mitigate bias in effect estimates (40).

### 4.2 Methodological limitations and potential improvements

Several methodological considerations warrant discussion. Our study utilized data from a single healthcare network (YRHIP) in eastern coastal China. While this network ensured comprehensive patient capture and reliable data quality, the single-center nature may limit result generalizability to other healthcare systems, particularly in regions with uneven resource distribution and varying clinical practices. Nevertheless, this study represents the first algorithm development for autoimmune disease identification using real-world data in China, establishing important theoretical and methodological foundations in this field. Future validation through multi-center databases could enhance algorithm applicability. Our algorithms primarily relied on diagnostic terminology and ICD-10 codes, aligning with typical EHR system structures and offering operational feasibility. However, for conditions requiring laboratory or imaging confirmation, such as RA, the inability to incorporate key biomarkers (rheumatoid factor, anti-cyclic citrullinated peptide antibodies) and imaging data may have limited sensitivity. Despite these constraints, our synthesis of outpatient, inpatient, and discharge records demonstrated meaningful improvements in algorithm performance, validating the importance of comprehensive data analysis approaches. Future studies could incorporate richer clinical data and employ advanced modeling methods to enhance diagnostic accuracy. The 2017 implementation of the real-name medical system introduced challenges in historical data linkage, potentially affecting both case identification accuracy. While we employed washout periods to mitigate these effects, future improvements in data linkage technologies and governance are needed to enhance analysis quality and reduce potential biases. Based on these considerations, future research directions should prioritize: establishing multi-center research networks to validate algorithms across diverse healthcare settings; exploring advanced methodologies for combining multi-source clinical data. These efforts will contribute to improving early diagnosis and ultimately enhance patient outcomes.

Our study has important implications for global public health. The validated algorithms provide valuable tools for conducting real-world studies in autoimmune diseases, particularly in resource-limited settings where comprehensive clinical assessments may be challenging to implement. By improving case identification through routinely collected electronic health data, these algorithms can support large-scale epidemiological surveillance across diverse populations. Furthermore, our findings may contribute to enhancing clinical practice by facilitating earlier identification of autoimmune conditions, potentially addressing diagnostic delays that are commonly observed among patients worldwide. Research indicates that patients with autoimmune diseases often experience diagnostic journeys averaging over 4.5 years, with multiple physician consultations before receiving a definitive diagnosis (43). By refining case identification algorithms, our work could aid clinical decision support systems in identifying potential autoimmune conditions earlier in the diagnostic process. This may help reduce the global disease burden through more timely interventions, which is particularly relevant given the progressive nature of many autoimmune conditions, where early treatment can significantly influence long-term outcomes (44). Notably, the algorithms we developed are rule-based, utilizing ICD codes and diagnostic terms, which may serve as a foundational framework for future research on more advanced approaches, such as model-based case identification algorithms.

## 5 Conclusion

This study presents the first comprehensive validation of identification algorithms for five major autoimmune diseases using EHR data in China. By combining diagnostic terminology and ICD-10 codes, we developed algorithms with satisfactory performance (PPV >70%) across most diseases, demonstrating their utility in real-world applications. For diseases such as Hashimoto’s thyroiditis and RA, high accuracy of the algorithms was achieved using single diagnostic terms or codes. However, the algorithms of more complex diseases like IBD and ITP need to improve sensitivity, highlighting the potential for further optimization by the inclusion of additional clinical biomarkers or advanced modeling techniques. These findings suggest two key areas for improvement: leveraging additional clinical parameters (such as laboratory biomarkers and imaging data) into algorithm development, and strengthening multi-center validation to enhance algorithm generalizability across diverse healthcare settings. The validated algorithms provide essential tools for future research in autoimmune disease epidemiology and pharmacovigilance studies, while emphasizing the importance of continued methodological refinement to support more accurate disease identification in real-world settings.

## Data Availability

The datasets presented in this article are not readily available because the dataset contains sensitive or private information, and confidentiality restrictions apply to ensure the protection of privacy. Requests to access the datasets should be directed to JTY, yangjunting@bjmu.edu.cn.
